# Expression of Bioinformatically Candidate miRNAs including, miR-576-5p, miR-501-3p and miR-3143, Targeting PI3K Pathway in Triple-Negative Breast Cancer

**DOI:** 10.31661/gmj.v8i0.1646

**Published:** 2019-11-10

**Authors:** Razie Hadavi, Samira Mohammadi-Yeganeh, Javad Razaviyan, Ameneh Koochaki, Parviz Kokhaei, Ahmadreza Bandegi

**Affiliations:** ^1^Department of Biochemistry, School of Medicine, Semnan University of Medical Sciences, Semnan, Iran; ^2^Student Research Committee, Semnan University of Medical Sciences, Semnan, Iran; ^3^Medical Nanotechnology Research Center, Shahid Beheshti University of Medical Sciences, Tehran, Iran; ^4^Department of Biotechnology, School of Advanced Technologies in Medicine, Shahid Beheshti University of Medical Sciences, Tehran, Iran; ^5^Department of Clinical Biochemistry, School of Medicine, Shahid Beheshti University of Medical Sciences, Tehran, Iran; ^6^Department of Molecular Biology and Genetic Engineering, Stem Cell Technology Research Center, Tehran, Iran; ^7^Cellular and Molecular Biology Research Center, Shahid Beheshti University of Medical Sciences, Tehran, Iran; ^8^Cancer Research Center and Department of Immunology, Semnan University of Medical Sciences, Semnan, Iran; ^9^Research Center of Physiology, School of Medicine, Semnan University of Medical Sciences, Semnan, Iran

**Keywords:** Triple Negative Breast Cancer, MicroRNA, PIK3CA, AKT1, Bioinformatics

## Abstract

**Background:**

Triple-negative breast cancer (TNBC) is an invasive and lethal form of breast cancer. PI3K pathway, which often activated in TNBC patients, can be a target of miRNAs. The purpose of this study was bioinformatic prediction of miRNAs targeting the key genes of this pathway and evaluation of the expression of them and their targets in TNBC.

**Materials and Methods:**

We predicted miRNAs targeting PIK3CA and AKT1 genes using bioinformatics tools. Extraction of total RNA, synthesis of cDNA and quantitative real-time polymerase chain reaction were performed from 18 TNBC samples and normal adjacent tissues and cell lines.

**Results:**

Our results demonstrated that miR-576-5p, miR-501-3p and miR-3143 were predicted to target PIK3CA, AKT1 and both of these mRNAs, respectively and were down-regulated while their target mRNAs were up-regulated in clinical samples and cell lines. The analysis of the receiver operating characteristic curve was done for the evaluation of the diagnostic value of predicted miRNAs in TNBC patients.

**Conclusion:**

The findings of our study demonstrated the reverse correlation between miRNAs and their target genes and therefore the possibility of these miRNAs to be proposed as new candidates for TNBC targeted therapies.

## Introduction

Breast cancer is one of the most prevalent neoplasms in women [1]. About 7.1 million women worldwide are diagnosed with this type of cancer and approximately 500,000 of them die from it annually [2]. Triple-negative breast cancer (TNBC) is a highly invasive and lethal form of breast cancer that does not represent any of estrogen receptors, progesterone receptors and human epidermal receptors 2 (HER2) [3, 4]. Concerning the fact that traditional breast cancer treatments are not so effective, modern and accessible therapeutic methods are required for treating this type of breast cancer. One of these developing methods is targeted therapies [5, 6]. miRNAs are small non-protein coding RNAs with an approximate length of 20-25 nucleotides. Binding of these molecules to 3′-untranslated regions (3′-UTR) of their target genes results in mRNA cleavage or translation repression [7-9]. In the past few years, different reports indicated a large number of miRNAs were involved in the pathophysiology of breast cancer. In general, the expression of tumor suppressor miRNAs can be significantly down-regulated whilst oncogenic miRNAs are up-regulated in breast cancer. Furthermore, different subtypes of this malignancy including TNBC show a specific expression profile of miRNAs [6, 10, 11]. Phosphatidylinositol 3-kinase (PI3K)/Akt/mTOR signaling, which is also called PI3K signaling pathway, promotes cell proliferation, survival, metabolism, and metastases and its aberrant activation is considered as a center for growth, survival and dynamics of cancer [6, 12-14]. PI3K from class IA of PI3Ks is the major role-player in this pathway. This enzyme phosphorylates phosphatidylinositol 4 and 5-bisphosphate (PIP2) to phosphatidylinositol 3, 4 and 5-triphosphate (PIP3). PIP3 activates AKT, a serine/threonine kinase, which affects the growth, survival and cell cycle of cancerous tissue [15-17]. Several studies have shown dysregulation of this pathway in triple-negative and other subtypes of breast cancer that leads to proliferation and cellular invasion [14]. Bioinformatics are up-to-dated and low-cost methods that absorbed a lot of attention today. These software tools and programs can predict miRNAs targeting a specific target gene in a short time based on biology and computer sciences [18]. To achieve more valuable results, several programs can be simultaneously utilized. One advantage of using these methods is to limit target sites for experimental verification [19-21]. Hence, the first purpose of the present study was to recruit bioinformatics for the prediction of miRNAs targeting PIK3CA and AKT1 mRNAs. Then, the expression of predicted miRNAs and their targets was investigated in breast cancer clinical samples and cell lines. The results of this study could be the starting point for further investigation of the diagnosis and treatment of TNBC.

## Material and Methods

### miRNAs Prediction

To predict miRNAs which target PI3K and AKT mRNAs, bioinformatic software applications including TargetScan [22], MicroCosm [23], DIANA Tool [24], miRanda [25], microPIR [26], miRTarBase [27], miRDB [28], miRWalk [29], miRNAMap [30], miRGator [31], miRSearch [32] were used.

### Primer Design for Investigating the Expression of Genes and miRNAs

mRNA sequences of target genes were received from GenBank, NCBI (www.ncbi.nih.nlm.gov). Then by Using Allele ID 7.0 and Oligo7 software, the best pair of forward and reverse primer was designed. Due to the small size of miRNAs, their expression analysis by conventional primer pairs is not possible. Therefore, the Stem-loop structures were used for cDNA synthesis based on the previously published studies [33, 34].

### Cell Culture

MDA-MB-231 and MCF-10A, as TNBC and normal breast cell lines respectively, were received from the National Cell Bank of Iran (Pasteur Institute of Iran, Tehran, Iran). MDA-MB-231 is an aggressive triple-negative breast ductal carcinoma cell line with estrogen, progesterone, and human epidermal growth factor receptors deficiencies. The normal breast cell line, MCF-10A, was used as control. MDA-MB-231 was cultured in Dulbecco’s Modified Eagle Medium (DMEM) including 10% fetal bovine serum (FBS) and 1% penicillin-streptomycin, and MCF-10A was cultured in DMEM containing 10% horse serum and 1% penicillin-streptomycin, at 37°C incubator with 5% CO2 and 98% humidity.

### Tumor and Normal Breast Clinical Samples

Eighteen clinical samples of triple-negative invasive ductal carcinoma and 18 adjacent normal tissues were received from the tumor bank of Imam Khomeini Hospital (Tehran, Iran). Samples were stored in RNA Later (Qiagen, Germany) in a -70°C freezer. The patients were not undergone previous treatments, including surgery, chemotherapy or radiotherapy. Written informed consent was taken from all participants. This study was under the supervision of the ethics committee of Semnan University of Medical Sciences (ethics code: IR.SEMUMS.REC.1394.120). The characteristics of breast cancer samples are presented in Table-1.

### Extraction of Total RNA and Synthesis of cDNA

The extraction of total RNA was performed by Gene All Hybrid-RTM purification kit (Korea) according to the protocol presented by manufacturer. Extracted RNA’s concentration was evaluated applying bio photometer (Eppendorf, Germany) and the quality of RNAs was investigated by gel electrophoresis. Then, synthesis of cDNA for genes and miRNAs was performed using Random hexamer and RT Stem-loop primers, respectively. and RevertAid® RT enzyme (Thermo scientific TM, USA) [33]. SNORD 47 and HPRT1 house-keeping genes were used in order to normalize the expression of miRNAs and target genes, respectively.

### Quantitative Real-Time Polymerase Chain Reaction (PCR)

Analysis of gene expression was performed in triplicates by Real-time PCR assay and the final volume was 13 μL, containing 4.5 μL dH2O, 1 μL cDNA (diluted at a ratio of 1/3), 0.5 μl of each reverse and forward primers (10 μM) and 6.5 μL RealQ Plus 2X Master Mix Green, High ROX^TM^ (Amplicon, Denmark).

Real-time PCR cycling profile for target and house-keeping genes was as follows: enzyme activation (95°C for 15 min), denaturation (95°C for 15 sec), annealing and extension (60°C for 1 min) for 40 cycles. At the end of amplification, analysis of melting temperature (Tm) was performed by raising the temperature from 60°C to 95°C by 0.2°C per sec increment. Analysis of predicted miRNAs by real-time PCR was done in triplicates with a total volume of 13 μL, containing 4.5 μL dH2O, 1 μL cDNA (diluted at a ratio of 1/3), 0.2 μL TaqMan® Probe (Macrogen, Korea), 0.4 μL for each reverse and forward primer (10 μM) and 6.5 μl RealQ Plus 2X Master Mix for Probe, High ROX^TM^(Amplicon, Denmark). Profile of real-time PCR reaction for target miRNAs and housekeeping was as follows: enzyme activation (95°C for 15 min), denaturation (95°C for 15 sec), annealing and extension (60°C for 1 min) for 40 cycles. The results were reported as Ct (Threshold Cycles). Finally, the increase or decrease in the level of the expressions was reported as fold changes.

### Receiver Operating Characteristic (ROC) Curve Analysis

For assessing the diagnostic value of predicted miRNAs, the area under curve (AUC) was calculated by ROC curve analysis. To achieve the best cut off point, the Youden index ([specificity + sensitivity]-1) was applied.

### Statistical Analysis

SPSS version 18 (SPSS, Inc., Chicago, IL, USA) and GraphPad Prism were used for statistical analysis and graph preparation, respectively. REST® 2009 was applied to analyze the results of Real-time PCR. This program applies ΔΔCT method for analysis. This software compares the expression level in groups of treated and control samples using iterations. Furthermore, the Mann-Whitney non-parametric test was used for comparing differences between tumor samples and normal surrounding tissues. Statistically, the P-value less than 0.05 was considered significant.

## Results

### Predicting miRNAs Targeting PIK3CA and AKT1

miRNAs were predicted applying bioinformatic software mentioned before. We arranged the results of TargetScan from the highest probable seed match 8mer to the lowest probable interactions. Then, miRNAs were investigated in other software and eventually miRNAs with the highest achieved-score were chosen. The hsa-miR-576-5p was selected as the miRNA targeting PIK3CA mRNA, hsa-miR-501-3p as the miRNA targeting AKT1, and hsa-miR-3143 as the miRNA targeting both of these mRNAs (Table-2). The bioinformatics analysis also revealed that the selected miRNAs had highest score and at least one binding site in the 3′-UTR of their target mRNAs ( Table-3).

### MiRNAs and Genes Expressions in MDA-MB-231

**Figure 1:**
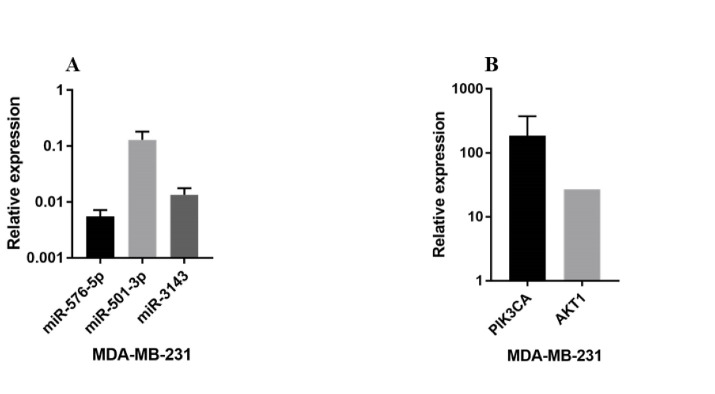
Expression of miRNAs and genes in cell lines; A: Down-regulation of miR-576-5p, miR-501-3p and miR-3143 in MDA-MB-231 cell line compared to MCF-10A; B: Up-regulation of PIK3CA, AKT1 in MDA-MB-231 cell line compared to MCF-10A.

Analysis of real-time PCR assay demonstrated the expression of miR-576-5p, miR-501-3p, and miR-3143 in the MDA-MB-231 cell line was decreased by 18.18, 9.4 and 7.4 folds, respectively in comparison with MCF-10A cells (Figure-1A). Our findings also indicated the expression of both PIK3CA and AKT1 genes were significantly increased by 185 and 26 folds, respectively in the MDA-MB-231 cell line compared to normal cells (Figure-1B). According to statistical analysis, the relative expression of all three miRNAs was statistically significant (P<0.05). Furthermore, the increase in the expression of target genes and the decline in the expression of miRNAs were significant between MDA-MB-231 and MCF-10A cell lines (P<0.05).

#### miRNAs and Genes Expressions in Clinical Specimens of Triple-Negative Invasive Ductal Carcinoma

**Figure 2:**
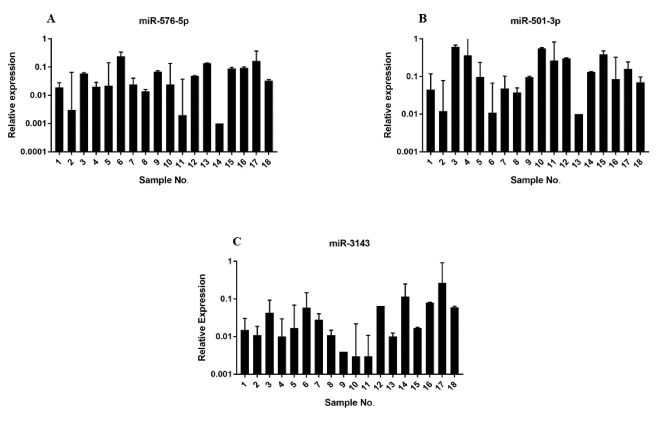
Down-regulation of miRNAs in tumor samples compared to normal samples; A: miR-576-5p, B: miR-501-3p, C: miR-3143.

**Figure 3:**
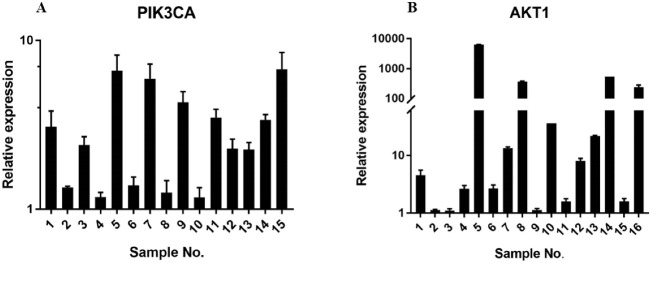
Relative expression of PIK3CA and AKT1 in TNBC clinical samples compared to normal adjacent tissues. A: Up-regulation of PIK3CA. B: Up-regulation of AKT1

Analysis of the miRNAs expression in 18 tumor samples compared with the tumor’s marginal tissue demonstrated that, interestingly, the expression of miR-576-5p, miR-501-3p, and miR-3143 decreased with an average of 16.9, 5.4, and 22 folds, respectively (Figure-2). The results also demonstrated that the expression of the PIK3CA gene in %83 of samples had an average increase of 3.1 folds (Figure-3A). The results also showed the expression of AKT1 in %83 of samples has an average increase of 82.5 folds and in one of the samples, it had been increased by even 6315 folds (Figure-3B). Furthermore, the relative expression of all genes and miRNAs was significant (P<0.05) in tumor samples compared to the normal ones.

### Diagnostic Value of miR-576-5p, miR-501-3p, and miR-3143 Expressions in TNBC

**Figure 4:**
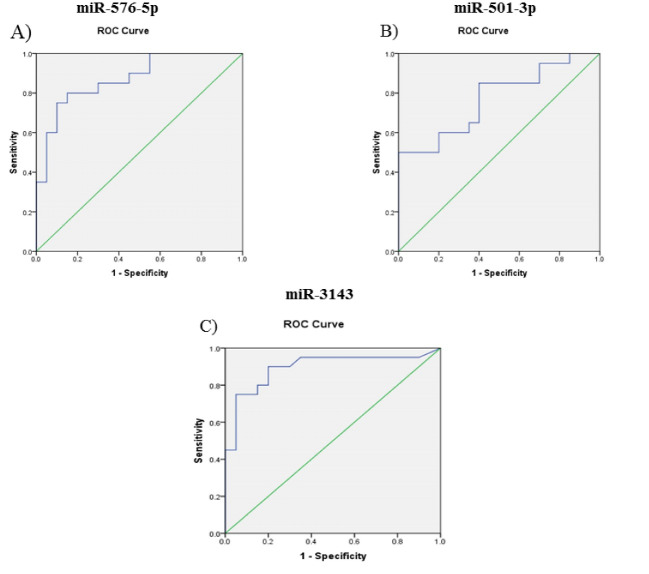
ROC curves of miRNAs including miR-576-5p (A) miR-501-3p (B) miR-3143 (C)

ROC curve analysis of miR-576-5p revealed that the AUC value was 0.872 (95% confidence interval [CI]=0.764- 0.981), and the best cut off point was 0.191 with the Youden index of 0.650, sensitivity of 80%, and specificity of 85%. For miR-501-3p, AUC value was 0.770 (95% CI=0.623-0.917), and the cutoff point of 0.178 with the Youden index of 0.450 was selected as the best. The sensitivity of 85% and specificity of 60% were obtained for this point. Eventually, the best-calculated cut off point for miR-3143 was 0.159 with Youden index of 0.650, sensitivity of 80%, and specificity of 85%. The AUC value of this miRNA was 0.894 (95% CI=0.785-1, Figure-4).

## Discussion

miRNAs are a large family of regulatory RNAs that cause post-transcriptional gene silencing. Changes in miRNAs expression profiles are one of the characteristics of different kinds of cancers including TNBC. TNBC, an aggressive subtype of breast cancer with poor prognosis, contains 15-20% of all cases of breast cancer. This type of breast cancer lacks specific markers for therapies, hence the main method in its treatment is chemotherapy. miRNAs-based targeted therapy is one of the developing new strategies [16, 35-39]. Concerning different pathways that are dysregulated in TNBC, PI3K/AKT/mTOR pathway was selected in this study due to its relation to the proliferation and survival of the patients. PI3K is one of the critical components of this pathway. PIK3CA gene that encodes catalytic α subunit of PI3K and also AKT1 gene, which is the downstream mediator of PI3K, were chosen as the target genes in the study [6, 12, 16, 38, 40, 41]. PIK3CA (catalytic subunit alpha of PI3K) is an oncogene located on chromosome 3q26.32 and is composed of 23 exons. AKT1 gene (AKT serine/threonine kinase 1) is an oncogene on chromosome 14q32.33 and is composed of 16 exons. Previous researches were inconsistent with our study and have shown up-regulation and the role of these two genes in cancers such as breast cancer. Cossu-Rocca *et al*. found that in 23.7% of TNBC cases, PIK3CA gene has been mutated. Therefore, PI3K pathway dysregulation and mutations in PIK3CA are commonly reported in TNBC [42]. The results of a study performed by Aleskandarany *et al*. showed that PIK3CA is an oncogenic biomarker, and its overexpression is significantly associated with the higher tumor grade, tumor invasion, axillary lymph node metastasis and vascular invasion in breast cancer patients. Therefore, it can consider a biomarker of breast cancer progression associated with poor prognosis. Also, in their study, 82.9 % of no special type case and 88.2% of medullary carcinoma showed overexpression of PIK3CA [43]. The results of the study performed by Ahmad *et al*. showed that AKT1 in breast cancer cell lines is overexpressed regardless of their estrogen receptor status [44]. Sun *et al*. found that the activity of AKT1 gene in breast, prostate, and ovarian cancers was increased, particularly in advanced tumors, and it has an important role in tumor development and progression [45]. Zhang *et al*. showed that miR-409-3p directly targets and inhibits AKT1 expression that leads to suppression of proliferation and invasion in breast cancer [46]. In this study, bioinformatics software and programs as mentioned in material and method part were used to predict miRNAs targeting PIK3CA and AKT1 genes. Finally, miR-576-5p and miR-501-3p, with the highest scores, were predicted as miRNAs targeting PIK3CA and AKT1 mRNAs, respectively. Moreover, miR-3143 was also predicted targeting both genes. To the best of our knowledge, there wasn’t any previous report about the role of these miRNAs in TNBC, except our previous in the parallel study that its results were in line with the current study and demonstrated down-regulation of these three miRNA in the MCF-7 cell line [47]. A study conducted on lung tumors by Mairinger *et al*. suggests that miR576-5p is significantly related to cell survival [48]. In the study of Dong *et al*., the miRNAs expression pattern in the peripheral blood of glioblastoma patients and normal cases were obtained by microarray. The results showed that the expression of miR-576-5p increased on average 3.05 folds in glioblastoma patients compared to healthy individuals [49] while in the study of Diaz-Prado *et al*. on patients with osteoarthritis, the expression of miR-576-5p in osteoarthritis chondrocyte pellets have been fallen 4.74 folds [50]. Soronen *et al*. have shown a significant increase in miR-576-5p expression in non-alcoholic fatty liver disease [51]. According to Haldrup *et al*. study, expression of miR-501-3p in serum and tumor samples of patients with prostate cancer has been increased [52]. In the case of miR-3143, the literature review also showed that there was only one article in PubMed referring to this miRNA. This study by Yilmaz *et al*. examined the expression of miR-3143 and some other miRNAs in the peripheral blood of Parkinson-suffering patients. By using real-time PCR method, they found that median expression value of miR-3143 was 0.4466 [53]. Our results showed that miR-576-5p, miR-501-3p, and miR-3143 were down-regulated in MDA-MB-231 and TNBC clinical specimens. ROC curve analysis showed that the declined expression of these miRNAs has a predictive value, i.e. AUC value of miR-3143 was nearly 0.9, which indicates the excellent discrimination between TNBC and normal samples. In addition, based on our findings, PIK3CA and AKT1 genes were up-regulated in TNBC samples and cell lines.

## Conclusion

The findings are in line with our hypotheses and indicate the possible negative correlation between the candidate miRNAs and their target genes. Given that PIK3CA and AKT1 genes promote proliferation and survival of tumor cells, their down-regulation by miRNAs results in a decline in the proliferation of cancerous cells. Therefore, miR-576-5p, miR-501-3p, and miR-3143 can be considered as novel candidates for miRNAs-based therapies in TNBC. Among these miRNAs, miR-3143, which targets both PIK3CA and AKT1 mRNAs and has the best discriminating value, can be the first suggestion. However, to practically confirm the effect of predicted miRNAs on their target genes further validation methods such as luciferase assay are needed.

## Acknowledgment

This project was funded by Semnan University of Medical Sciences, Semnan, Iran (grant number: 909) and Shahid Beheshti University of medical sciences, Tehran, Iran (grant number: 13442). The authors should thank Stem Cell Technology Research center, Tehran, Iran, and molecular Biology Research Center, Shahid Beheshti University of Medical sciences, Tehran, Iran for providing technical help.

## Conflict of Interest

Not declared.

**Table 1:** Investigated Parameters of Breast Cancer Samples (N=18)

Variable	Abundance	Percentage
Age (year) ≤35 35-50 >51		
	3	17
	7	39
	8	44
Grade I II III		
	2	11
	2	11
	14	78
Lymphatic invasion + -		
	9	50
	9	50
Vascular invasion + -		
	10	56
	8	45
Perineural invasion + -		
	6	33
	12	67

**Table 2:** Prediction of miRNAs Predicting PIK3CA and AKT1

**Gene**	**miRNA**	**TargetScna**	**MicroCosm**	**DIANA Tool- microT-CDS**	**DIANA Tools- TarBase**	**miRanda**	**microPIR**	**miRTarBase**	**miRDB**	**miRWalk**	**miRNAMap**	**miRGator**	**miRSearch**	**SUM**
PIK3CA	hsa-miR-576-5p	1	1	1	0	1	1	0	0	1	0	1	0	7
AKT1	hsa-miR-501-3p	1	1	0	0	1	1	0	0	1	0	0	0	5
PIK3CA	Has-miR-3143	1	0	1	0	1	0	0	0	1	0	0	0	4
AKT1	Has-miR-3143	1	0	0	0	1	0	0	0	0	0	0	0	2

**Table 3:** miRNAs Binding Site on 3′-UTR of Target mRNAs

**Predicted miRNA**	**Position of AKT1 3′ UTR pair to miRNA**	**Site type**
miR-501-3p	Position 90-96: 5′…GUUUUUAUUUCUCGGGUGCAUU … 3′	7mer-m8
miR-3143	Position 276-283: 5′… UCCGAUUCACGUAGGGAAAUGUUAA... 3′	8mer
**Predicted miRNA**	**Position of PIK3CA 3′ UTR pair to miRNA**	**Site type**
miR-576-5p	Position 109-115: 5′…CAAUCCAUGAACAGCAUUAGAAU … 3′	7mer-m8
	Position 2380-2386: 5′…AACCAAAUUAAUUCUAUUAGAAG … 3′	7mer-m8
	Position 3015-3022: 5′…UUAUAUUAAAAGCCCAUUAGAAA … 3′	8mer
	Position 5460-5466: 5′… UGGUAUAACUGCAUUCAUUAGAAG... 3′	7mer-m8
miR-3143	Position 443-449: 5′… AGUAAACUGGAGUUUAUGUUAAA... 3′	7mer-A1
	Position 909-915: 5′…GAAAAUAGAAGUAUUAAUGUUAU... 3′	7mer-m8
	Position 90-96: 5′…UGCUUGAUUUUAGGUAUGUUAAU... 3′	7mer-A1
